# The effects of P2Y_12_ adenosine receptors’ inhibitors on central and peripheral chemoreflexes

**DOI:** 10.3389/fphys.2023.1214893

**Published:** 2023-07-19

**Authors:** Stanislaw Tubek, Piotr Niewinski, Anna Langner-Hetmanczuk, Maksym Jura, Wiktor Kuliczkowski, Krzysztof Reczuch, Piotr Ponikowski

**Affiliations:** ^1^ Institute of Heart Diseases, Wroclaw Medical University, Wroclaw, Poland; ^2^ Institute of Heart Diseases, University Hospital, Wroclaw, Poland; ^3^ Department of Physiology, Wroclaw Medical University, Wroclaw, Poland

**Keywords:** ticagrelor, clopidogrel, carotid body, peripheral chemoreflex, central chemoreflex, dyspnea, P2Y12 adenosine receptor inhibitors

## Abstract

**Introduction:** The most common side effect of ticagrelor is dyspnea, which leads to premature withdrawal of this life-saving medication in 6.5% of patients. Increased chemoreceptors’ sensitivity was suggested as a possible pathophysiological explanation of this phenomenon; however, the link between oversensitization of peripheral and/or central chemosensory areas and ticagrelor intake has not been conclusively proved.

**Methods:** We measured peripheral chemoreceptors’ sensitivity using hypoxic ventilatory response (HVR), central chemoreceptors’ sensitivity using hypercapnic hyperoxic ventilatory response (HCVR), and dyspnea severity before and 4 ± 1 weeks following ticagrelor initiation in 11 subjects with chronic coronary syndrome undergoing percutaneous coronary intervention (PCI). The same tests were performed in 11 age-, sex-, and BMI-matched patients treated with clopidogrel. The study is registered at ClinicalTrials.com at NCT05080478.

**Results:** Ticagrelor significantly increased both HVR (0.52 ± 0.46 vs. 0.84 ± 0.69 L min^-1^ %^−1^; *p < 0.01*) and HCVR (1.05 ± 0.64 vs. 1.75 ± 1.04 L min^−1^ mmHg^−1^; *p < 0.01*). The absolute change in HVR correlated with the change in HCVR. Clopidogrel administration did not significantly influence HVR (0.63 ± 0.32 vs. 0.58 ± 0.33 L min^-1^%^−1^; *p = 0.53*) and HCVR (1.22 ± 0.67 vs. 1.2 ± 0.64 L min^−1^ mmHg^−1^; *p = 0.79*). Drug-related dyspnea was reported by three subjects in the ticagrelor group and by none in the clopidogrel group. These patients were characterized by either high baseline HVR and HCVR or excessive increase in HVR following ticagrelor initiation.

**Discussion:** Ticagrelor, contrary to clopidogrel, sensitizes both peripheral and central facets of chemodetection. Two potential mechanisms of ticagrelor-induced dyspnea have been identified: 1) high baseline HVR and HCVR or 2) excessive increase in HVR or HVR and HCVR. Whether other patterns of changes in chemosensitivities play a role in the pathogenesis of this phenomenon needs to be further investigated.

## Introduction

Ticagrelor is one of the first-line antiplatelet agents prescribed following acute coronary syndrome (ACS) (Valgimigli et al., 2018). The efficacy of the drug in the secondary prevention of cardiovascular events is more pronounced when compared to that of clopidogrel (Wallentin et al., 2009); however, the relative risk of its premature discontinuation due to side effects is also significantly higher (approximately 59% greater) (Arora et al., 2019).

Dyspnea, the most common side effect of ticagrelor, was present in up to 38.6% of individuals enrolled in several clinical studies (Storey et al., 2010; Cattaneo and Faioni, 2012; Parodi and Storey, 2015). While this phenomenon does not influence the efficiency of ticagrelor and cardio-pulmonary function of recipients, it leads to treatment cessation in up to 6.5% of studied patients (Wallentin et al., 2009; Storey et al., 2010; Storey et al., 2011a; Bonaca et al., 2016). The dyspnea-related risk of ticagrelor withdrawal is 6.4 times higher than that of clopidogrel (Arora et al., 2019). Moreover, dyspnea in post-ACS patients requires prompt and usually costly diagnostic measures (Parodi and Storey, 2015).

It is well-established that ticagrelor-induced dyspnea 1) is not the consequence of pulmonary dysfunction (Storey et al., 2010; Storey et al., 2011b), 2) is dose-dependent (Husted et al., 2006; Cannon et al., 2007), and 3) relies on ticagrelor plasma levels (Ortega-Paz et al., 2018). However, the precise pathogenesis of this side effect is unknown.

Two hypothetical mechanisms have been proposed as the explanation of ticagrelor-related dyspnea. Cattaneo et al. suggested that the local adenosine accumulation and/or cumulation of intracellular cAMP may lead to afferent pulmonary parasympathetic C-fiber activation and, as a result, to dyspnea sensation (Cattaneo and Faioni, 2012). Another pathomechanism was suggested by Giannoni et al., who found an elevated ventilatory response to hyperoxic hypercapnia (central chemoreceptors’ hypersensitivity) and normoxic hypercapnia in patients taking ticagrelor compared to those on prasugrel (Giannoni et al., 2021). The hypersensitivity was greater in patients with dyspnea (Giannoni et al., 2021) and resolved following drug withdrawal in the case reported (Giannoni et al., 2016), which suggests the possible role of chemoreflex in its pathogenesis, but does not clearly delineate the contribution of isolated peripheral and central chemosensory areas.

Human chemoreceptors are localized in two major anatomical locations: 1) in the bifurcation of common carotid arteries (carotid bodies) and along the aorta and its main branches (aortic bodies)—these are known as arterial or peripheral chemoreceptors (PCh) (Kumar and Prabhakar, 2012); 2) in the brain stem and the diencephalon—known as central chemoreceptors (CCh) (Nattie and Li, 2012; Li et al., 2013). PCh are sensitive to the decrease in O_2_ and increase in H^+^ or CO_2_ arterial blood concentrations. Otherwise, CCh are responsive to an increase in H^+^ or CO_2_ within cerebrospinal fluid (Marshall, 1994; O'Regan and Majcherczyk, 1982; Guyenet et al., 2010; Nattie and Li, 2012). Stimulation of both groups of chemoreceptors leads to hyperventilation and sympathoexcitation (Nattie and Li, 2012; Marshall, 1994; O'Regan and Majcherczyk, 1982). Moreover, the activation of chemoreceptors undoubtedly leads to dyspnea sensation (Whipp, 1994; Buchanan and Richerson, 2009).

In our previous studies, we described the stimulatory properties of adenosine on peripheral chemoreceptors (PCh) in humans (Tubek et al., 2016a). It has also been reported that P2Y_12_ receptors are present in mammalian chemosensory cells (glomus cells) and that activation of these receptors inhibits hypoxia-induced response (Agarwal et al., 2011). Thus, both actions of ticagrelor—P2Y_12_ adenosine receptor’s inhibition and adenosine re-uptake blockage—may be involved at the peripheral chemoreceptors’ level (Husted et al., 2006; Armstrong et al., 2014), leading to their stimulation or/and hypersensitivity, which results in dyspnea sensation.

However, the influence of orally administered P2Y_12_ adenosine receptor inhibitors on PCh or CCh has not been studied so far. We hypothesize that these pharmacological agents, particularly ticagrelor, may increase the sensitivity of chemoreceptors. PCh would be primarily affected since ticagrelor does not cross the blood–brain barrier (Brott et al., 2014). Because sensitization of PCh is known to augment central chemoreflex gain, one may speculate that CCh functioning would also be affected by ticagrelor (Dahan et al., 2007; Dahan et al., 2008).

To address those issues, we designed the proof-of-the-concept study to investigate, for the first time, the effects of clopidogrel and ticagrelor initiation on the sensitivity of PCh and CCh.

## Methodology

### Studied population

The study was approved by the local Ethics Committee (Komisja Bioetyczna, Wroclaw Medical University) and was performed in accordance with the latest review of the Helsinki Declaration. All subjects gave informed written consent. The study is registered at ClinicalTrials.com at NCT05080478.

Patients suffering from chronic coronary artery disease referred to the Cardiology Department of University Hospital in Wroclaw for elective percutaneous coronary intervention (PCI) were prospectively screened to find two clinical scenarios: 1) patients taking clopidogrel in the peri-PCI period and planned to be switched to ticagrelor following the PCI, due to the complexity of the procedure (according to the 2017 ESC focused update on dual antiplatelet therapy in coronary artery disease, such a change may be considered in high-thrombotic, low-bleeding risk patients); 2) P2Y_12_ inhibitor-naïve patients who were planned to be administered clopidogrel in the peri-PCI period. Eleven patients from the first group constituted the ticagrelor group. These subjects were age-, BMI-, and gender-matched with 11 individuals from the second group (clopidogrel group).

Subjects excluded from study participation included those older than 80 years, scheduled for coronary artery bypass grafting following coronary angiography, prescribed with a strong cytochrome P-450 3A inhibitor or inducer, as well as patients with contraindications for ticagrelor or clopidogrel; untreated clinically significant bradycardia; clinically significant anemia and/or thrombocytopenia; any severe valvular heart disease requiring further interventional or surgical treatment; symptomatic bronchial asthma or severe chronic obstructive pulmonary disease; heart failure in NYHA IV class; any mental disorder that may influence patient compliance; end-stage renal failure on hemodialysis; pregnancy or breastfeeding; carotid artery stenting or carotid endarterectomy in the past medical history. Additionally, menstruating women or those undergoing hormone replacement therapy were excluded from the trial because of the possible influence of gonadocorticoids on the measured parameters (Tatsumi et al., 1997). Being aware of the potential effects of the other cardiovascular medication on PCh or CCh, we also excluded patients whose medications, other than P2Y_12_ inhibitors, were altered during the study timeframe (Langner-Hetmańczuk et al., 2022). Detailed information regarding subjects’ demographic data is shown in Table 1.

**TABLE 1:** Subjects’ demographic data and baseline parameters. Values are presented as mean ± SD.

	Ticagrelor n = 11	Clopidogrel n = 11	*P*-value
Age [y]	63 ± 6	64 ± 3	0.69
Sex [male/female]	8/3	8/3	1.0
BMI [kg (m^2^) ^−1^]	28.6 ± 4.2	28 ± 4.8	0.78
LVEF [%]	59 ± 5	57 ± 11	0.95
NTproBNP [pg ml^−1^]	170 ± 130	151 ± 140	0.78
Hb [g%]	14.2 ± 1.4	14.3 ± 1.2	0.51
GFR [ml min^−1^]	108 ± 24	98 ± 30	0.39
Medical history			
Myocardial infarction	4 (36%)	5 (45%)	0.5
Ischemic stroke	0	1 (9%)	0.5
Hypertension	9 (81%)	8 (73%)	0.5
HFrEF	0	1 (9%)	0.5
Diabetes	3 (27%)	3 (27%)	1.0
CCS class prior to PCI [median]	II	II	0.83
Active smoking	3 (27%)	2 (18%)	0.5
Therapy			
Aspirin	11 (100%)	11 (100%)	1.0
B-blockers	10 (91%)	10 (91%)	1.0
ACEI/ARB	11 (100%)	11 (100%)	1.0
Statins	11 (100%)	11 (100%)	1.0
Ezetimibe	3 (27%)	0	0.11
Calcium blockers	2 (18%)	4 (36%)	0.32
Trimetazidine	1 (9%)	1 (9%)	1.0
Ivabradine	0	1 (9%)	0.5
Diuretics	1 (9%)	5 (45%)	0.07
Aldosterone antagonists	0	2 (18%)	0.24
Metformin	2 (18%)	2 (18%)	1.0
SGLT2 inhibitor	3 (27%)	2 (18%)	0.5
Gliclazide	1 (9%)	1 (9%)	1.0
Baseline parameters			
HVR [l min^−1^ %^−1^]	0.52 ± 0.46	0.63 ± 0.32	0.21
SBPR [mmHg %^−1^]	1.03 ± 0.62	0.7 ± 0.3	0.25
HRR [bpm %^−1^]	0.47 ± 0.25	0.28 ± 0.14	0.13
HCVR [l min^−1^ mmHg^−1^]	1.05 ± 0.64	1.22 ± 0.67	0.55
HR [bpm]	62 ± 9	63 ± 8	0.84
SBP [mmHg]	132 ± 15	128 ± 27	0.08
DBP [mmHg]	69 ± 12	68 ± 13	0.58
VI [l min^−1^]	10.2 ± 3.7	10.5 ± 2.9	0.47
SpO_2_ [%]	96 ± 1	97 ± 1	0.77
ETCO_2_ [mmHg]	39 ± 5.2	37 ± 4.7	0.75

BMI, body mass index; LVEF, left ventricle ejection fraction; NTproBNP, N-terminal prohormone of brain natriuretic peptide; Hb, hemoglobin level; GFR, creatinine clearance according to the Cockcroft–Gault equation; HFrEF, heart failure with reduced ejection fraction; CCS, Canadian Cardiovascular Society scale; PCI, percutaneous coronary intervention; ACEI, angiotensin-converting enzyme inhibitor; ARB, angiotensin receptor blocker; SGLT2, sodium/glucose cotransporter 2; HVR, hypoxic ventilatory response; SBPR, systolic blood pressure response to hypoxia; HRR, heart rate response to hypoxia; HCVR, hypercapnic hyperoxic ventilatory response; HR, heart rate; SBP, systolic arterial pressure; DBP, diastolic arterial pressure; VI, minute ventilation; SpO_2_, oxygen saturation; ETCO_2_, end-tidal carbon dioxide.

### Study protocol

**FIGURE 1:**
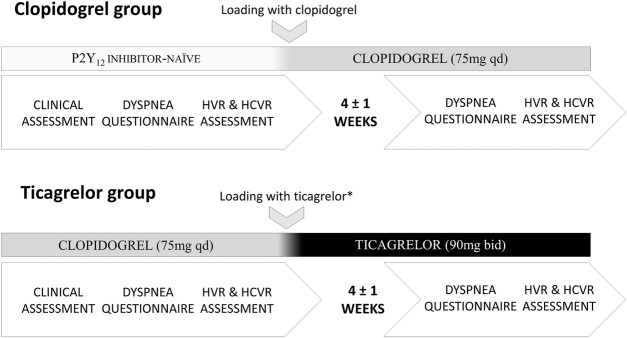
Study protocol (HVR–hypoxic ventilatory response; HCVR–hypercapnic hyperoxic ventilatory response; * the switch took place 1–2 days following PCI).

Both groups of patients underwent the same protocol consisting of 1) clinical assessment (laboratory testing and transthoracic echocardiography), 2) dyspnea assessment (questionnaire), 3) evaluation of baseline hemodynamic and ventilatory parameters, 4) measurement of the sensitivity of PCh, and 5) measurement of the sensitivity of CCh. The clinical assessment took place during the hospitalization scheduled for PCI. Procedures 2–5 were performed during a 2-hour-long session, with an approximately 30-min break between PCh and CCh sensitivity testing. The procedures were performed twice: 1) prior to the switch from clopidogrel to ticagrelor and 4 ± 1 weeks later in the ticagrelor group and 2) before clopidogrel loading dose and 4 ± 1 weeks later in the clopidogrel group. Study participants were asked to avoid caffeine intake or nicotine on the day of testing and to take the P2Y12 inhibitor approximately 4 h prior to the second session. The latter was based on the pharmacokinetics of ticagrelor, clopidogrel, and their active metabolites (Husted et al., 2012; Karaźniewicz-Łada et al., 2014). Further details are presented in Figure 1.

### Assessment of baseline hemodynamic and ventilatory parameters

Subjects were examined in a quiet room with a stable temperature of 22 °C in the supine position. After being attached to the study equipment via a silicone mask covering the mouth and nose (Hans Rudolph, Inc, Shawnee, KS, United States), patients were allowed to rest for 5 to 10 min to get familiarized with the research environment. After this period, baseline values were averaged from the 5 min of the recording preceding PCh sensitivity testing. A one-way open breathing circuit (Hans Rudolph, Inc, Shawnee, KS, United States) with an inspiratory arm connected to a high-pressure electric valve, which allowed silent switching between 100% nitrogen and room air, and the expiratory arm connected via a 1000 L min^−1^ flowhead (MLT3000L, ADInstruments) to a differential pressure transducer (FE141 Spirometer, ADInstruments, Sydney, Australia) was used for the measurement of minute ventilation (VI). Hemodynamic parameters were monitored non-invasively, in a beat-by-beat manner, using a Nexfin device (BMEYE B.V, Amsterdam, Netherlands) and included heart rate (HR), systolic arterial blood pressure (SBP), and diastolic blood pressure (DBP). Blood oxygen saturation (SpO_2_) was evaluated using a pulse oximeter (Radical-7, Masimo Corporation Irvine, CA, United States) with an ear clip, and the recording was shifted backward by 15 s to compensate for the circulatory delay. All data were collected using PowerLab 16/30 (ADInstruments, Dunedin, New Zealand) and recorded on a laptop (Dell Inc, Round Rock, TX, United States).

### Assessment of peripheral chemosensitivity—hypoxic ventilatory response (HVR)

We used the well-established poikilocapnic intermittent hypoxia method to calculate the hypoxic ventilatory response (HVR), as a measure of PCh sensitivity [L min^−1^ %^−1^] (Chua and Coats, 1995). Subjects attached to the aforementioned study equipment breathing room air were silently switched to 100% nitrogen for 10–35 s, which caused decrease in SpO_2_ with minimal values in the range between 90% and 70%. Hypoxic exposures, of randomized lengths, were repeated 4 to 8 times per test. Following each nitrogen administration, patients were asked to rest between 3 and 5 min until the measured parameters returned to baseline levels. The single ventilatory response was calculated as an average from the three largest consecutive breaths following individual nitrogen administration. Data from the 90 s preceding each gas administration were used as the baseline. HVR was expressed as the slope of the linear regression describing the relationship between all artifact-free, single ventilatory responses and the associated nadirs of SpO_2_, including the baseline values of VI and SpO_2_. Isocapnia was not artificially maintained during the test. Data were blinded and analyzed by the researcher who was not directly involved in data collection.

### Assessment of hemodynamic response to hypoxia

The HR and SBP responses to hypoxia were calculated simultaneously with the HVR. The hemodynamic data were smoothed by taking a moving average based on a 3-s moving window and then a weighted average of a 200-ms window centered on each data point. The second technique was achieved by convolving the original signal with a Gaussian filter built from 201 sample points with a variance of 100. The peak HR and peak SBP following each successful nitrogen administration were plotted against the corresponding nadir of SpO_2_. The slope of the linear regression, including the baseline data, was calculated similarly to the HVR calculation for HR–heart rate response to hypoxia (HRR [bpm %^−1^]) and SBP–systolic blood pressure response to hypoxia (SBPR [mmHg %^−1^]) (Niewinski et al., 2013). In two subjects (one from each group), non-invasive recording of good-quality hemodynamic data was not possible due to frequent ventricular extrasystoles or finger deformations secondary to osteoarthritis.

### Assessment of central chemosensitivity—hypercapnic hyperoxic ventilatory response (HCVR)

Read’s rebreathing technique—hypercapnic hyperoxic ventilatory response (HCVR)—was employed to calculate CCh sensitivity [L min^−1^ mmHg^−1^] (Read, 1967). Subjects sitting in an upright position were attached via the silicone mask (Hans Rudolph, Inc, Shawnee, KS, United States) and the heated linear pneumotach (Series 3813, Hans Rudolph, Inc, Shawnee, KS, United States) to a three-way balloon valve (Series 8250, Hans Rudolph, Inc, Shawnee, KS, United States), allowing for the switch between room air and the 6-L bag filled initially with 100% oxygen. The pneumotach was connected to a differential pressure transducer (FE141 Spirometer, ADInstruments, Sydney, Australia). ETCO_2_ (end-tidal carbon dioxide) was monitored with a CO_2_ analyzer attached to the silicone mask (CapStar 100, CWE). The test started with a 5-minute-long resting phase when subjects were breathing in room air for familiarization with the study equipment, followed by a rebreathing phase. During the test, VI and ETCO_2_ were measured breath-by-breath until the patient signaled breathlessness or ETCO_2_ exceeded 70 mmHg. Mean EtCO_2_ levels at the beginning of rebreathing phase were as follows: 38.3 ± 4 vs. 37.7 ± 3.5 mmHg and 39.1 ± 4.3 vs. 37.3 ± 4.4 mmHg for ticagrelor vs. clopidogrel and before/after drug initiation, respectively; all *p* = NS. Due to the hyperoxic nature of HCVR testing, mean SpO2 during the rebreathing phase was 100% for all groups and time points. An inflection point was identified by visual inspection of the chart relating VI to ETCO_2_, and the HCVR was calculated as a slope of the data past this point (MacKay et al., 2016). Data were blinded and analyzed by the researcher who was not directly involved in the data collection.

#### Assessment of dyspnea

Dyspnea was evaluated using an investigator-designed questionnaire consisting of 1) visual–analog scales assessing the presence and the severity of rest and exertional dyspnea; 2) six multiple choice questions allowing for the characterization of the dyspnea sensation: the frequency, duration, timing, onset, course, and accompanying symptoms of the episodes (the original version of the questionnaire is provided in Supplementary Material). Each case of dyspnea was carefully assessed by an experienced physician to exclude secondary causes of the symptom, e.g., bronchospasm, congestion, cardiac ischemia, and additional blood, or diagnostic tests (e.g., echocardiography) were performed when required. The ticagrelor-related dyspnea was identified when all the following criteria were met: manifestation following the P2Y_12_ inhibitor initiation, independence of exertion, lack of any clinical signs, and symptoms suggestive of other known pathology causing new onset dyspnea. This definition was in line with that of previous studies on the subject (Storey et al., 2011a; Cattaneo and Faioni, 2012).

### Data and statistical analysis

Statistica 13 (StatSoft Inc.), LabChart 8 (ADInstruments), and MATLAB (MathWorks) were employed to analyze the data. The distribution of the variables was tested using Shapiro–Wilk’s W test. Normal distribution was found for HCVR, age, BMI, glomerular filtration rate (GFR), HR, SBP, DBP, VI, SpO_2_, ETCO_2_, and change (delta) in HRR; all other variables were non-normally distributed. The unpaired Student’s t-test or Mann–Whitney U test was used where appropriate to assess the differences between studied groups. The significance of changes in measured parameters following P2Y_12_ inhibitor initiation within studied populations was evaluated using the paired Student’s t-test and the Wilcoxon paired data test. The differences in categorical data were assessed using Fisher’s exact test. The correlations between variables were assessed with Pearson correlation coefficient, followed by visual graph evaluation for potential non-linear relationships. Data are presented as mean and standard deviation (SD). A *p-value* < 0.05 was considered statistically significant.

## Results

### Baseline hemodynamic, ventilatory and demographic data

No significant differences in baseline hemodynamic and ventilatory parameters were found neither between groups nor within studied populations when baseline data prior to each HVR testing were compared (all *p = NS*). Furthermore, baseline HVR, SBPR, HRR, and HCVR before switch from clopidogrel to ticagrelor in the ticagrelor group and before initiation of clopidogrel in P2Y_12_ naïve individuals in the clopidogrel group, (Figure 1) were not significantly different. Baseline data are presented in Table 1.

#### The influence of clopidogrel on chemoreflexes

**FIGURE 2:**
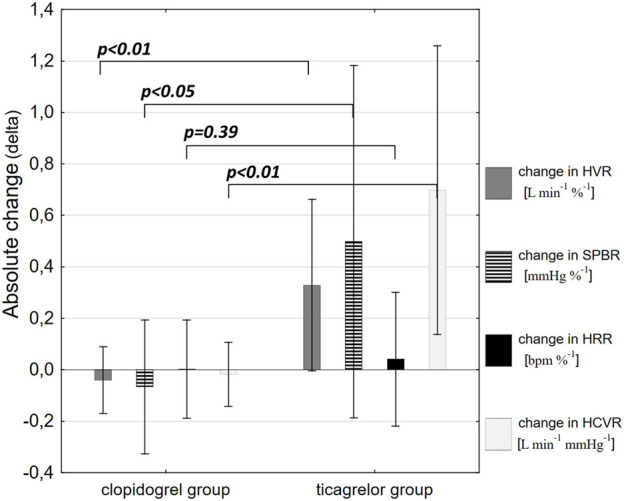
Comparison of post-drug changes between the study groups. Data are presented as mean absolute change from the baseline (delta) in hypoxic ventilatory response (HVR), systolic blood pressure response to hypoxia (SBPR), heart rate response to hypoxia (HRR), and hypercapnic hyperoxic ventilatory response (HCVR).

**FIGURE 3:**
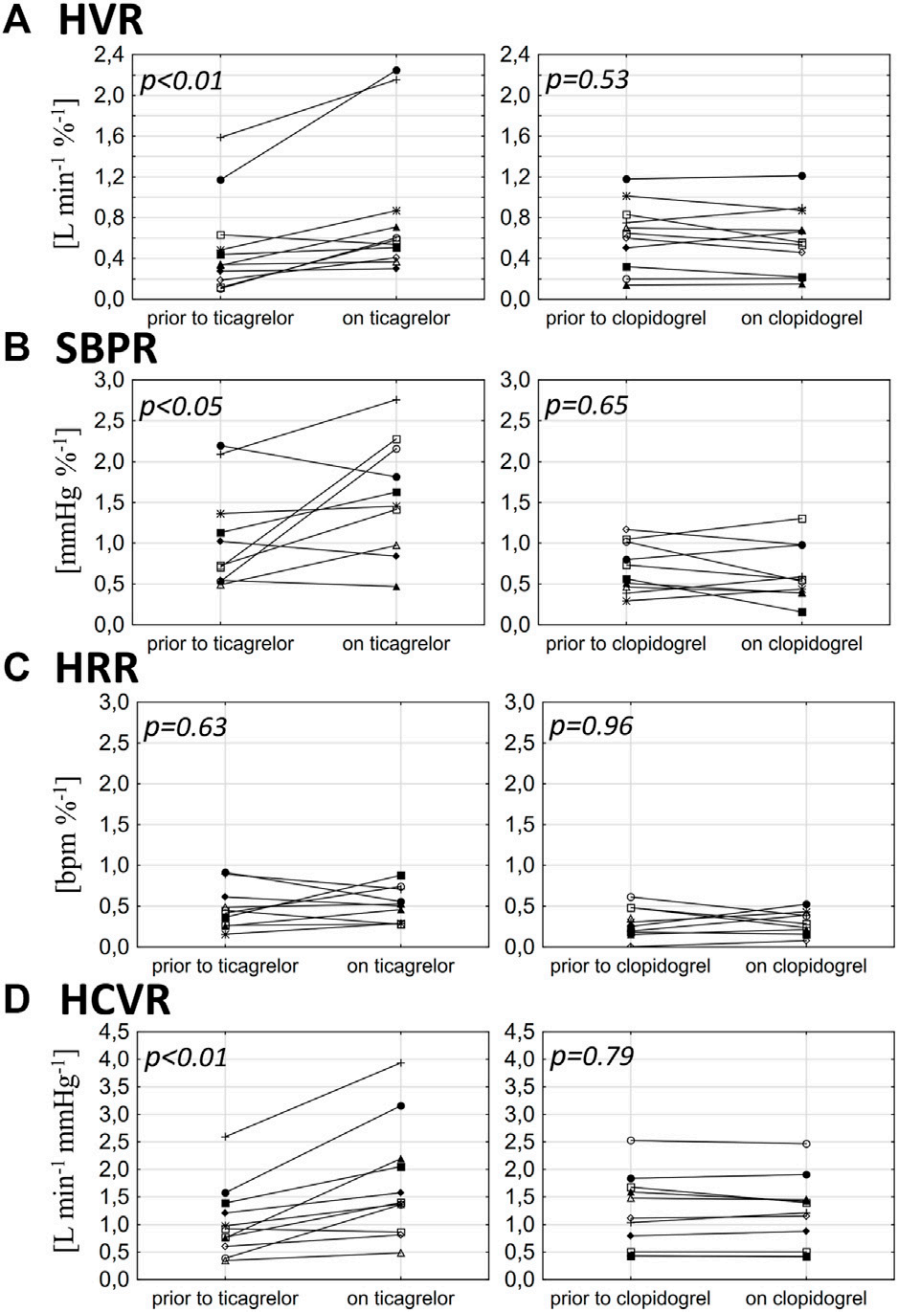
Influence of ticagrelor and clopidogrel on hypoxic ventilatory response (HVR) **(A)**, systolic blood pressure response to hypoxia (SBPR) **(B)**, heart rate response to hypoxia (HRR) **(C)**, and hypercapnic hyperoxic ventilatory response (HCVR) **(D)** in particular individuals.

Clopidogrel administration did not significantly influence any of the measured parameters, including HVR (0.63 ± 0.32 vs. 0.58 ± 0.33 L min^−1^ %^−1^; *p = 0.53*), SBPR (0.7 ± 0.3 vs. 0.71 ± 0.41 mmHg %^−1^; *p = 0.65*), HRR (0.3 ± 0.18 vs. 0.28 ± 0.14 bpm %^−1^; *p = 0.96*), and HCVR (1.22 ± 0.67 vs. 1.2 ± 0.64 L min^−1^ mmHg^−1^; *p = 0.79*). The mean absolute change from the baseline (delta) in chemosensitivities and the comparison of post-drug changes between study groups are presented in Figure 2. Individual data are presented in Figure 3.

#### The influence of ticagrelor on chemoreflexes

**FIGURE 4:**
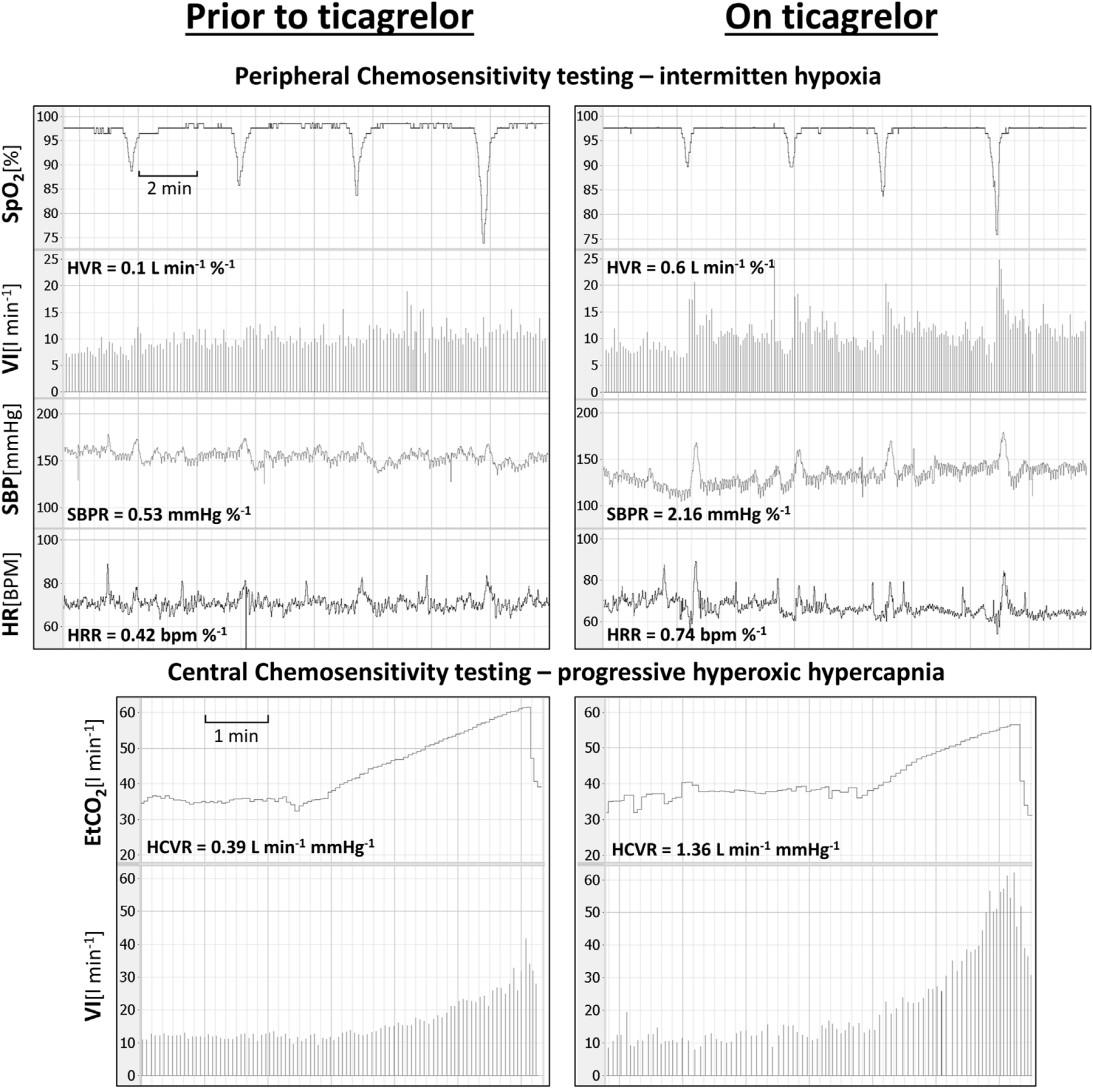
Raw data from central and peripheral chemosensitivity testing in one of the subjects experiencing ticagrelor-related dyspnea (SpO_2_—oxygen saturation; VI–minute ventilation; SBP–systolic blood pressure; HR–heart rate; ETCO_2_—end-tidal carbon dioxide).

Ticagrelor significantly increased HVR (0.52 ± 0.46 vs. 0.84 ± 0.69 L min^−1^ %^−1^; *p < 0.01*), SBPR (1.03 ± 0.62 vs. 1.58 ± 0.7 mmHg %^−1^; *p < 0.05*), and HCVR (1.05 ± 0.64 vs. 1.75 ± 1.04 L min^−1^ mmHg-1; *p < 0.01*), but did not influence HRR (0.47 ± 0.25 vs. 0.52 ± 0.21 bpm %^−1^; *p = 0.63*). The mean absolute change from the baseline (delta) in chemosensitivities and the comparison of post-drug changes between study groups are presented in Figure 2. Individual data are presented in Figure 3. Raw data from central and peripheral chemosensitivity testing in one of the subjects with dyspnea are presented in Figure 4.

#### Relationship between chemoreflex changes in the ticagrelor group

**FIGURE 5:**
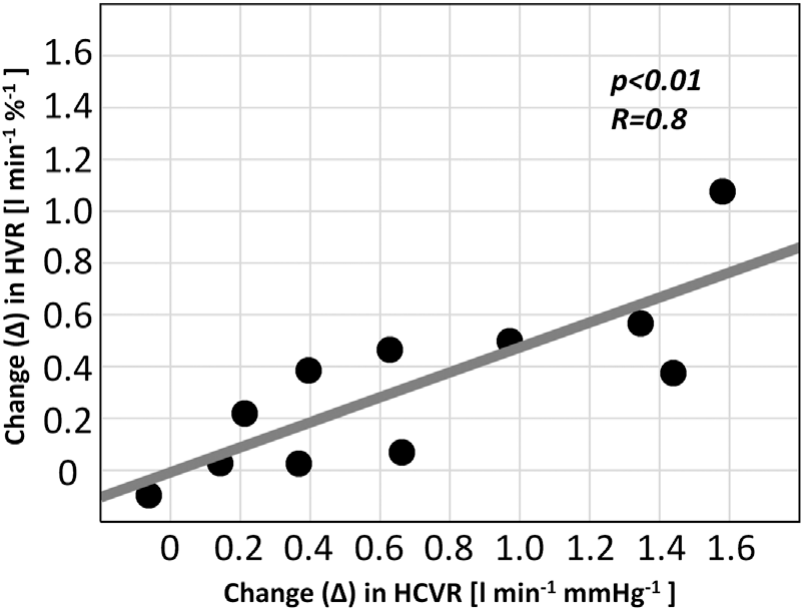
Relationship between chemoreflex changes in patients receiving ticagrelor (HVR–hypoxic ventilatory response; HCVR–hypercapnic hyperoxic ventilatory response).

The absolute change in the HVR correlated with the absolute change in the HCVR in the ticagrelor group (R = 0.8; *p < 0.01*)—Figure 5. No correlation was found for the change in the HVR and the change in the HRR and SBPR (all *p = NS*). There were no relationships between the values of baseline HVR or baseline HCVR and the change in HVR or HCVR (all *p = NS*).

#### Prevalence of dyspnea in studied populations

Eight subjects (five in the clopidogrel group and three in the ticagrelor group) reported dyspnea prior to the study intervention. In seven of eight patients (four in the clopidogrel group and three in the ticagrelor group), the sensation was moderate to severe (5.7 ± 3.2/10) and was provoked mainly by physical exertion. During the follow-up visit, after the PCI, those subjects declared alleviation of dyspnea. In those patients, the initial dyspnea was interpreted as a symptom of coronary artery disease. One of eight subjects (from the clopidogrel group) reported resting and exercise-related dyspneic episodes of moderate severity (6/10), occurring several times a day, lasting several hours, alleviating spontaneously or following tranquilizer administration. The symptoms were also present following the clopidogrel initiation and the PCI, and hence were judged to be of psychogenic origin.

New onset dyspnea following P2Y_12_ inhibitor initiation was reported by three subjects in the ticagrelor group (27% of ticagrelor-treated subjects) and by none in the clopidogrel group. None of those subjects reported shortness of breath prior to PCI. Dyspnea was described as mild by two patients (3/10 and 4/10) and as severe by one subject (8/10). Dyspneic episodes occurred suddenly and spontaneously on a daily basis, most frequently in the evenings, and alleviated gradually. In two patients with mild dyspnea, the events terminated in the 2^nd^ and the 3^rd^ week of ticagrelor treatment. In those subjects, the therapy remained uninterrupted. In the remaining severely dyspneic subject, who had high baseline HVR and HCVR, the sensation persisted for more than 6 weeks, and the switch to clopidogrel was made at the patient’s request. Detailed characteristics of the dyspneic subjects compared to non-dyspneic individuals can be found in Table 2. Briefly, we found that patients with ticagrelor-related dyspnea presented either with a marked relative increase in HVR (5- to 6-fold compared to the baseline, which was accompanied with a 3.5-fold increase in HCVR in one case) or were characterized by high both HVR and HCVR at the baseline with modest additional sensitization following ticagrelor.

**TABLE 2:** Characteristics of subjects presenting with ticagrelor-related dyspnea.

	Subject # 1	Subject # 2	Subject # 3	Non-dyspneic subjects [mean ± SD]
Dyspnea				
Severity	Mild (4/10)	Mild (3/10)	Severe (8/10)	-
Duration (weeks)	Transient (2)	Transient (3)	Persistent (6^a^)	-
Age [y]	66	60	64	63 ± 7
Sex [male/female]	Female	Male	Male	
BMI [kg (m^2^) ^−1^]	28.2	34.9	27.1	28 ± 4
LVEF [%]	60	52	60	59 ± 5
Baseline parameters				
HVR [l min^−1^ %^−1^]	0.1	0.11	1.59	0.48 ± 0.31
HCVR [l min^−1^ mmHg^−1^]	0.39	0.77	2.6	0.97 ± 0.41
HR [bpm]	71	55	57	62 ± 9
SBP [mmHg]	154	110	132	133 ± 14
DBP [mmHg]	88	64	67	67 ± 11
VI [l min^−1^]	7	18.3	8.9	9.7 ± 2.8
SpO_2_ [%]	97	96	94	96.5 ± 1
ETCO_2_ [mmHg]	37	37	32	40 ± 5
Change in HVR [l min^−1^ %^−1^]/[%]^b^	0.5/480	0.47/401	0.57/36	0.26 ± 0.37/50 ± 53
Change in HCVR [l min^−1^ mmHg^−1^]/[%]^b^	0.97/249	0.62/81	1.34/52	0.59 ± 0.61/60 ± 60

^a^ Ticagrelor was switched to clopidogrel on the patient’s request.

^b^ Absolute/relative values.

BMI, body mass index; LVEF, left ventricle ejection fraction; HVR, hypoxic ventilatory response; SBPR, systolic blood pressure response to hypoxia; HRR, heart rate response to hypoxia; HCVR, hypercapnic hyperoxic ventilatory response; HR, heart rate; SBP, systolic arterial pressure; DBP, diastolic arterial pressure; VI, minute ventilation; SpO_2_, oxygen saturation; ETCO_2_, end-tidal carbon dioxide.

## Discussion

In this pilot study, we followed unique clinical scenarios to investigate the influence of clopidogrel and ticagrelor treatment on HVR and HCVR. There are several novel findings of the study: 1) ticagrelor and clopidogrel exert divergent influence over the HVR and HCVR; 2) ticagrelor significantly increases ventilatory (HVR) and blood pressure (SBPR) responses to selective PCh stimulation with transient hypoxia, when the effects of clopidogrel are negligible; 3) ticagrelor (but not clopidogrel) significantly increases ventilatory response to selective CCh stimulation (HCVR) with progressive hyperoxic hypercapnia; 4) there is a strong relationship between the changes in HVR and HCVR following ticagrelor initiation.

As mentioned before, the influence of ticagrelor on chemoreflex has been previously proposed by Giannoni et al., who observed elevated HCVR in post-ACS patients taking ticagrelor compared to those on prasugrel (Giannoni et al., 2021). However, it is not clear whether it was 1) the result of higher baseline (before the drug initiation) sensitivity or 2) the result of the augmentation of the reflex response caused by the drug. Moreover, the peri-ACS period is a difficult time for the investigation of the influence of P2Y12 inhibitors on chemoreflex. ACS is an emergency and life-threatening state, which usually needs instant treatment; hence, the assessment of baseline chemoreflex (before the drug initiation) could be very challenging (Ibanez et al., 2017; Collet et al., 2020). Moreover, ACS in the acute phase may itself lead to augmentation of PCh sensitivity, as described in an animal model by Rocha et al. (2003). Finally, the majority of ACS patients are prescribed with multiple new medications (other than P2Y_12_ inhibitors) potentially influencing the chemoreflex arc, including B-blockers and renin–angiotensin–aldosterone axis inhibitors (Giannoni et al., 2021; Langner-Hetmańczuk et al., 2022). To overcome described confounding factors, we examined patients with chronic coronary disease on stable drug regimens (apart from the P2Y_12_ inhibitor introduction). Such a clinical course allowed for the assessment of chemosensitivities before and during clopidogrel or ticagrelor *de novo* administration.

Revising available literature, we identified two potential actions of ticagrelor, which may be responsible for peripheral chemoreflex sensitization: 1) inhibition of adenosine P2Y_12_ receptors on chemosensory (glomus) cells (Husted et al., 2006) and 2) inhibition of cellular adenosine re-uptake (Armstrong et al., 2014).

P2Y_12_ adenosine receptors are present on mammalian glomus cells in PCh, and stimulation of these purinergic receptors leads to inhibition of hypoxia-induced response (Agarwal et al., 2011). Hence, the blockade of P2Y_12_ receptors with ticagrelor may lead to hyperreactivity and prolonged activation of chemosensory cells. However, clopidogrel, which is also a P2Y_12_ receptor inhibitor, does not exert such an effect (Cannon et al., 2007; Storey et al., 2010). This can be explained by the different pharmacokinetics of these antiplatelets. Ticagrelor is a reversible P2Y_12_ receptor antagonist since constant, high serum concentrations have to be maintained to keep the receptors deactivated (Husted et al., 2006). Otherwise, clopidogrel is an irreversible P2Y_12_ receptor antagonist, administered once a day, which activates the metabolite’s serum half-life ranging between 7 and 8 h (Plosker and Lyseng-Williamson, 2007). This is enough to inhibit platelet function; however, it does not affect nucleated cells (including chemosensory glomus cells of the PCh) because of their ability to restore the active surface proteins when the serum levels of clopidogrel decrease between the doses (Cattaneo and Faioni, 2012). This observation could be confirmed by a consistently higher risk of dyspnea in patients treated with other reversible P2Y_12_ inhibitors such as cangrelor and elinogrel, when compared to clopidogrel (Harrington et al., 2009; Welsh et al., 2012).

The second uniqueness to ticagrelor action, which may lead to dyspnea sensation, is adenosine re-uptake inhibition with secondary elevation in tissue and serum adenosine concentrations (Armstrong et al., 2014; Bonello et al., 2014; Ortega-Paz et al., 2018). It has already been shown in animals that adenosine directly augments afferent carotid sinus nerve signaling (Chen et al., 1997; Conde et al., 2008) and independently potentiates the activating effect of hypoxia on chemosensory cells (Conde et al., 2017). Finally, the stimulating properties of adenosine on human PCh were already confirmed in our previous studies—adenosine solution injected directly into the common carotid artery caused hyperventilation similar to that provoked by hypoxia (Tubek et al., 2016b).

On the other hand, some of the ticagrelor actions may at least theoretically reduce PCh sensitivity. For example, increased adenosine levels may also cause vasodilatation with consequent PCh hyperemia. Inversely, the hypoperfusion of PCh is known to increase chemosensitivity by acting on shear stress sensitive transcription factors (Ding et al., 2011; Layland et al., 2014). It was also shown that compared to clopidogrel, ticagrelor better improves endothelial function and more effectively reduces the generation of reactive oxygen species (Schultz et al., 2015; Campo et al., 2017). Similarly, P2Y12 receptor antagonists are known to reduce inflammation, with some data suggestive of more pronounced effects of ticagrelor than of clopidogrel and prasugrel (Husted et al., 2010; Jeong et al., 2017; Huang et al., 2021; Adali et al., 2022). Given the fact that inflammatory stress and oxidative stress are well-defined PCh stimulants, the aforementioned actions of ticagrelor should result in diminished reflex response (Iturriaga et al., 2022). Nevertheless, as was clearly shown in our study, the stimulatory properties of ticagrelor dominate the overall response from PCh.

The influence of ticagrelor on the HCVR presents as a more complex issue, as chemosensory neurons of CCh are exposed only to the cerebrospinal fluid and are separated from the blood by the blood–brain barrier (Guyenet et al., 2010; Nattie and Li, 2012). Ticagrelor and adenosine do not cross the blood–brain barrier, which makes direct interactions unlikely (Isakovic et al., 2004; Brott et al., 2014). Thus, one could hypothesize that the sensitization of CCh seen in our study may be secondary to the increased PCh sensitivity. Such causality has been reported in numerous previous studies in animal (Blain et al., 2010; Smith et al., 2015) and human models (Dahan et al., 2007; Dahan et al., 2008). The notion of sensitization of CCh *via* increased reflex response from PCh following ticagrelor administration is further supported by the significant correlation between the change in HVR and HCVR observed in our study.

Ticagrelor-related dyspnea was present in three of 11 (27%) patients included in the study, which is concomitant with the reports from previous trials (Storey et al., 2010; Cattaneo and Faioni, 2012; Parodi and Storey, 2015). No drug-related dyspnea was reported in the clopidogrel group. The case-by-case analysis of the subjects presenting ticagrelor-related dyspnea revealed two potential patterns of the changes in chemosensitivities (Table 2): 1) low baseline HVR, followed by an excessive increase (5–6-fold) in response to ticagrelor (present in two of three subjects); 2) high baseline chemosensitivities (approximately 3-fold higher compared to non-dyspneic subjects) with less prominent increase after drug administration (one of three subjects). Subjects with the former pattern reported mild and self-limiting dyspnea; on the contrary, the individual characterized by the latter pattern presented with severe, chronic dyspnea, which did not resolve until drug cessation. One cannot exclude that the third pattern, namely, high baseline chemosensitivities with subsequent excessive increase following ticagrelor administration, also exists, but that was not observed in our study. Moreover, in one of the dyspneic subjects with low baseline chemosensitivities, a 6-fold increase in HVR was accompanied with a 3.5-fold increase in HCVR following ticagrelor initiation. Interestingly, an increase in HVR and HCVR was also found in non-dyspneic patients (the mean relative change was +50 ± 53% for HVR and +60 ± 60% for HCVR). Further studies are required to investigate the pathophysiological background of ticagrelor-related dyspnea in larger samples of patients.

Treatment with ticagrelor increased the ventilatory response (HVR) and also augmented the blood pressure response (hypertension) to the hypoxic stimulus (SBPR) but did not influence HRR. This finding is concomitant with those of our previous observations and further suggests that PCh (at least carotid bodies) are not responsible for hypoxia-mediated tachycardia. As we previously reported, selective activation of the carotid body in conscious humans leads to bradycardia instead of tachycardia (Tubek et al., 2016b), and bilateral resection of the carotid bodies diminishes HVR and SBPR but does not influence HRR (Niewinski et al., 2014). However, the HRR levels could be underestimated in our study since most of the subjects were treated with medications acting on the cardiovascular system, including B-blockers, which may mask the changes in the parameter.

It is worth noticing that hyperreflexia of chemoreceptors is known to play a role in the pathogenesis of sleep-disordered breathing and sympathetically mediated diseases, such as hypertension, and is an ominous sign in patients with systolic heart failure (Ponikowski et al., 1999; Ponikowski et al., 2001; Abdala et al., 2012; Dempsey et al., 2012; Ribeiro et al., 2013; Schultz et al., 2013). Giannoni et al. already described Cheyne–Stoke’s breathing pattern in dyspneic patients receiving ticagrelor, which abated after drug cessation (Giannoni et al., 2016). While ticagrelor-related dyspnea itself is regarded as a benign condition, sleep-disordered breathing is well-known to have a harmful influence on the cardiovascular system, including the increased risk of arterial hypertension, arrhythmia, or cardiomyopathy development (Wolk et al., 2003). Periodic breathing is also a well-defined predictor of poor prognosis in heart failure patients (Lanfranchi et al., 1999). Whether the long-term use of ticagrelor, with consequent chemoreceptor oversensitivity, may lead to the development of other (apart from periodic breathing) diseases caused by chronic activation of chemoreceptors, such as hypertension, or may worsen the prognosis of heart failure patients needs to be studied. There are some limitations of the present study. First, the number of dyspnea cases is low in our group, which makes the conclusive description of the pathomechanism behind this side effect rather problematic; however, our study was designed as a proof-of-concept project and, as such, might be used for power calculation for future trials with greater number of participants. Second, we did not assess ticagrelor and its metabolite’s serum concentrations, but all the patients received the same dose of ticagrelor (90 mg twice a day) and took the drug approximately 4 h prior to chemosensitivity testing. Third, the assessment of dyspnea was based on the self-reported questionnaire performed before and after the drug intervention, which may result in over-reporting of this subjective sensation. Fourth, poikilocapnic intermittent hypoxia was employed to test HVR in the studied population, which may lead to an underestimation of the reported changes in PCh sensitivity due to transient hyperventilation-induced hypocapnia following hypoxic exposures. Fifth, patients in the ticagrelor group were pre-treated with clopidogrel before switching to ticagrelor. Although clopidogrel does not influence chemoreflexes and the baseline HVR and HCVR did not differ between groups, an impact of this dissimilarity on the results cannot be completely excluded. Sixth, we did not perform long-term respiratory monitoring to reveal disordered breathing in our population. Nevertheless, as discussed earlier, this issue has been extensively investigated by Giannoni et al., who already described the association between ticagrelor treatment, elevated HCVR, and sleep-disordered breathing (Giannoni et al., 2021). Seventh, the beat-by-beat hemodynamic measurements were not possible in two of 22 subjects included in the study (one from the ticagrelor group and another from the clopidogrel group). This might lead to underestimation of the differences in hemodynamic responses to hypoxia.

Finally, the chronic coronary artery disease clinical model was used in our study to assess the effects of P2Y12 inhibitor initiation on chemoreceptors. It is rather unlikely, as our results are concomitant with those made by Giannoni et al., 2021, that the effects of the drug would be different in ACS patients. This is why we believe that the results could be extrapolated to all ticagrelor recipients, including those following ACS.

## Conclusion

Ticagrelor administration is associated with a significant increase in peripheral and central chemoreceptor sensitivities, while clopidogrel does not exert such effects. Analysis of the individual, numerical data of subjects suffering from ticagrelor-related dyspnea suggests that two mechanisms may be involved in the pathogenesis of the phenomenon–one is an excessive increase in PCh sensitivity or PCh and CCh sensitivities and the other one is the high baseline (before the drug administration) PCh and CCh sensitivities. The second pattern was observed in a participant with severe ticagrelor-induced dyspnea. Further studies are necessary to shed more light on the pathomechanism of ticagrelor-related dyspnea. This will allow for 1) development of treatment strategies to alleviate this common side effect; 2) inventing screening tests, to identify patients at risk for ticagrelor-induced dyspnea; and 3) tailoring P2Y_12_ inhibitor therapy by choosing drugs with lower potential for chemosensitization with unbeneficial consequences.

## Data Availability

The raw data supporting the conclusion of this article will be made available by the authors, without undue reservation.

## Abbreviations

PCh, peripheral chemoreceptors; CCh, central chemoreceptors; HVR, hypoxic ventilatory response; SBPR, blood pressure response to hypoxia; HRR, heart rate response to hypoxia; HCVR, hypercapnic hyperoxic ventilatory response.
