# Supplementary material for: The effects of P2Y12 adenosine receptors’ inhibitors on central and peripheral chemoreflexes
Source: Front Physiol. 2023 Jul 19;14:1214893. doi: 10.3389/fphys.2023.1214893 (PMC10394699; doi:10.3389/fphys.2023.1214893)
Supplement: Supplementary file 1 [file DataSheet1.pdf]

Dyspnea Questionnaire

Following questions are related to your perception of well-being in the last 4 weeks.

1. Please put a point on the scale below in a spot corresponding to your **dyspnea perception on exercise**.

No dyspnea at all

Dyspnea at minimal exercise/rest

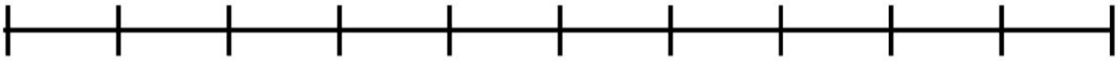

2. Please put a point on the scale below in a spot corresponding to your **dyspnea perception at rest**.

No dyspnea at all

Dyspnea all the time

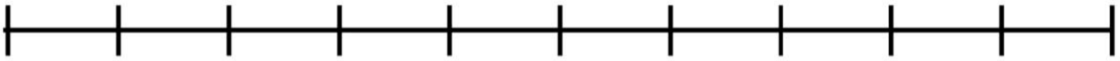

3. Please put a point on the scale below in a spot corresponding to **the severity of the worst dyspnea episode at rest**.

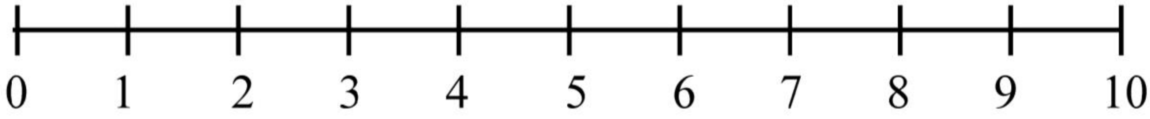

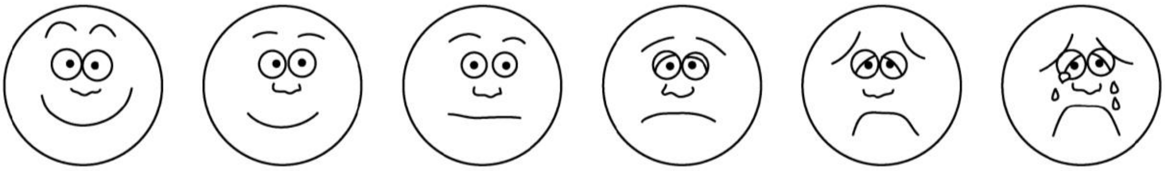

Following questions concern DYSPNOA AT REST, not dyspnoea at exertion.

1. How often did you feel dyspnoeic at rest?
    a. I have no dyspnea at rest
    b. Less than once a week
    c. 1-2 times per week
    d. 3-4 times per week
    e. Every day
    f. Several times per day

If you have chosen answer a. in the first question you can stop filling the questioner, but if you have chosen one of the answers from b. to f. please follow to questions 2.-6.:

2. How long did episodes of dyspnea last on average?
    a. A few minutes
    b. Approximately half an hour
    c. Approximately one hour
    d. Few hours
    e. Whole day
3. What time did episodes of dyspnea appear usually?
    a. In the mornings (6.00-9.00)
    b. Before noon (9.00-12.00)
    c. In the afternoons (12.00-17.00)
    d. In the evenings (17.00-22.00)
    e. At nights (22.00-6.00)
    f. Each time after drugs administration
    g. I felt dyspnoeic all the time
4. The onset of dyspnoeic episodes was usually:
    a. Sudden and quick
    b. It rose slowly and the beginning was difficult to define
5. Dyspnoeic episodes appeared usually:
    a. Without any reason
    b. With cough
    c. With palpitations
    d. With a rise in blood pressure
    e. In supine position and disappeared when sitting/standing
    f. In supine position and did not disappear when sitting/standing
    g. On exercise and did not disappear after rest
6. During the episodes dyspnea sensation was:
    a. Fairly constant
    b. Its intensity was changing: it was rising and diminishing without any reason
    c. After sudden beginning its intensity was diminishing until it disappeared
    d. It was rising on exercise and diminishing at rest
    e. It was rising in supine position and diminishing when sitting/standing

Additional comments:

……
……
……
……
……
